# Grape Seed Proanthocyanidin Extract Improved some of Biochemical Parameters and Antioxidant Disturbances of Red Blood Cells in Diabetic Rats

**Published:** 2015

**Authors:** Esrafil Mansouri, Layasadat Khorsandi, Maasoumeh Zare Moaiedi

**Affiliations:** a *Department of Anatomical Sciences, Cellular and Molecular Research Center, Faculty of Medical Sciences, Ahvaz Jundishapur University of Medical Sciences, Iran. *; b *Laboratory of Amir Kabir Hospital, Ahvaz, Iran.*

**Keywords:** GSPE, Biochemical factors, Erythrocyte antioxidant enzymes

## Abstract

Grape seed proanthocyanidin extract (GSPE) has a broad spectrum of biologic properties against oxidative stress. This study aimed to investigate the effects of GSPE on biochemical factors and antioxidant enzymes of erythrocyte in diabetic rats. Diabetes was induced through single injection of streptozotocin (50 mg.Kg^-1^, *i.p*). Forty Male Sprague-Dawley rats were divided into four Groups: Group 1, healthy control group; Group 2, healthy group treated with GSPE (200 mg.Kg^-1^); Group 3, diabetic control group; Group 4, diabetic group treated with GSPE (200 mg.Kg^-1^) for 4 weeks. At the end, the experimental animals were sacrificed and blood samples were collected and plasma parameters and erythrocytes antioxidant status were evaluated. The results show, treatment with GSPE significantly reduced *(P*<0.001*)* urine volume, proteinuria and biochemical factors such as blood urea nitrogen, creatinine, triglyceride, total cholesterol, low density lipoprotein and very low density lipoprotein as well as malondialdehyde. Also GSPE treatment significantly (*P*<0.005) increased high density lipoprotein, total protein and albumin. Moreover GSPE significantly increased antioxidant enzymes activity such as: superoxide dismutase, glutathione peroxidase and catalase. These results suggest that GSPE can ameliorate biochemical abnormalities and antioxidant system status in streptozotocin- induced diabetic rats probably by its potent antioxidant features.

## Introduction

Diabetes mellitus (DM) is an important metabolic disorder. Due to the increasing over the world incidence of diabetes mellitus creates a financial burden for global public health (1). It is distinguished with hyperglycemia and long-term complications which involves the nerves, kidneys, eyes and blood vessels. Although the mechanisms causing diabetic complications are still unknown, but more attention has been focused to the function of oxidative stress. It has been suggested that oxidative stress may be involved in the development of various diabetic complications (2, 3). Moreover, diabetes is associated with many features such as, increased lipid peroxidation (4), change of the glutathione redox situation, and decreased activity of antioxidant enzymes. These alterations show that oxidative stress induced by hyperglycemia (5). Several defense systems participate against oxidative damages caused by diabetes. One of these system are antioxidants destroy free radicals (6). Nowadays to minimize oxidative damage, there is an increasing interest to use natural antioxidants. The researchers showed that many of damaging effects of oxidative stress are reduced with intake of dietary antioxidant such as vitamins and other nonfood antioxidants such as flavonoids and polyphenols (7). Proanthocyanidin is a compound extracted from grape seed and its basic structure unit is the catechin. Proanthocyanidins are containing monomer, dimer and trimer catechin, all of which are water-soluble molecules and contain a number of phenolic hydroxyls (8). Polyphenolic compounds having a very important function of antioxidant, they can clean off the free radicals, and reduce the membrane lipid peroxidation, so they can reduce the occurrence of free radical-related diseases and delay aging (9, 10). Current researches have revealed that grape seed proanthocyanidin extract can clear off free radicals, protect the over-oxidative damage caused by free radicals, (11, 12) and prevent a range of diseases caused by free radicals, such as myocardial infarction, atherosclerosis, drug-induced liver and kidney injury; moreover, it has functions of anti-thrombotic, anti-tumor, anti-mutagenic, anti-radiation, and anti-fatigue (13-15). Therefore, we investigated the effects of grape seed proanthocyanidin extract (GSPE) on some biochemical parameters and antioxidant enzymes in blood of diabetic rats.

## Experimental

*Animals and experimental design*

This study was reviewed and approved by the Ethics Committee of Ahvaz University of Medical Sciences. Forty male Sprage- Dawley rats (150-170 g) were prepared from animal house central of Ahvaz Jundishapur University of Medical Sciences (AJUMS). All animals were housed in cages with 12/12 h light/dark cycle at 21 ± 2 ^◦^C. The animals were fasted overnight and diabetes was induced by a single intraperitoneal injection STZ (50 mg/Kg body weight) (Sigma- U.S.A.) a freshly dissolved in citrate buffer (o.1 M pH 4.5), while control rats were injected with vehicle buffer only. Blood samples were obtained from the tail vein of the animals at 72 hours after STZ injection and fasting blood glucose levels were determined with a glucose strip test in a glucometer (Easy Gluco Blood Glucose Monitoring system, Infopia, Korea). Rats with fasting blood glucose levels above 250 mg/dl were used as the diabetic animals. The treatment was started on the fourth day after the STZ injection and this was considered as the first day of treatment. The treatment was continued daily for 4 weeks. The rats were divided into four Groups of comprising 10 animals in each group as follows: Group 1, healthy control group; Group 2, healthy group treated with GSPE (200 mg.Kg^-1^); Group 3, diabetic control group; Group 4, diabetic group treated with GSPE (200 mg.Kg^-1^). Rats were orally administered with GSPE (Hangzhou Joymore Technology Co., *Ltd* China) dissolved in normal saline. At the end of 4^th^ week six rats selected randomly from every investigated group then rats were kept individually in metabolic cages to collect 24 hour urine for measurements of urine output and excreted protein. Assessment of excreted protein was done by using commercially available kit on spectrophotometer (Pars Azmon, Iran). Then rats were scarified under ether anesthesia and their blood was collected and processed 

*Preparation of erythrocyte lysate*

Whole blood was obtained by cardiac puncture and collected into heparinized tubes. Erythrocytes were separated from plasma by centrifugation at 3500 rpm for 10 min. To get packed erythrocytes, the remaining erythrocytes were washed tree or four times with an isotonic solution of NaCl (0.9%) until a colorless supernatant was observed. To obtain erythrocyte hemolysate, 500 µL packed erythrocyte was crushed by addition of four volumes of cold redistilled water. Then was centrifuged twice to remove all of the cell membranes: first for 10 minutes in the tube centrifuge at 3500 rpm at 4 °C, then in an Eppendorf centrifuge at 7800 rpm for 5 minutes at 4 °C. Clear supernatant was obtained as hemolysate. plasma was used for the determination of biochemical parameters and malondialdehyde (MDA) and hemolysate used for study of antioxidant status.

*Biochemical parameters*

Biochemical factors such as fasting blood sugar (FBS), total protein (TP), albumin (Alb), blood urea nitrogen (BUN), creatinine (Cr), and lipid profile study such as, triglycerides (TG), cholesterol (Chol), high density lipoprotein (HDL), low density lipoprotein (LDL) and very low density lipoprotein (VLDL) were determined by auto analyzer (Vita lab Selectra E, Netherland) in Golestan Hospital, Ahvaz using commercially available kits (Pars Azmon, Iran).

*Estimation of MDA in plasma*

MDA was assayed according to the method of Satho (16). 0.5 mL of plasma was mixed with 1.5 mL trichloroacetic acid (TCA) (10%) and was centrifuged (4000× g for 10 minutes) then 1.5 mL of supernatant was blended with 2 mL thiobarbituric acid (TBA) (0.67%) and was kept for 30 minutes at 100 °C. After cooling, 2 mL *n*-butanol was added to the solution and then centrifuged at 4000× g for 15 min, finally pink supernatant absorbance was read at wavelengths 535 nm. Values were expressed as nmol/mL. As 99% of the TBARS is malondialdehyde (MDA), TBARS concentrations of the samples were calculated from a standard curve using 1,1,3,3-tetramethoxypropane.

*Determination of antioxidant enzymes activity in erythrocyte lysate*

Superoxide dismutase (SOD) and Glutathione peroxidase (GPX) activities were determined using the diagnostic kits RANSOD and RANSEL produced by RANDOX (Randox Labs, Crumlin, UK) and were expressed in unit per gram of haeomglobin (Hb).

Catalase (CAT) activity was measured by the method of Aebi (17). The final reaction volume of 1 mL contained 50 mM potassium phosphate (pH 7.0), 19 mM H_2_O_2_, and a 20-50 µL sample. The reaction was initiated by the addition of H_2_O_2_, and absorbance changes were measured at 240 nm (20 °C) for 30 s. Catalase activity was estimated by using the molar extinction coefficient of 43.6M^-1^ cm^-1 ^for H_2_O_2_. The level of CAT was expressed in terms of µmoles H_2_O_2_ consumed/min per gram of haeomglobin.

*Statistical analysis*

Data are expressed as the mean ± SD. Statistical significance of differences was assessed with one-way analysis of variance (ANOVA) by SPSS for Windows version 18 (IBM, USA) followed by Tukey´s -test. P<0.05 was assumed as statistically significant.

## Results

*Changes in urine volume (UV), urinary protein 24-hour (UP24h) and biochemical parameters*

As shown in the Table 1, urine volume (UV) and UP24h were higher in group 3 when compared with the group 1 (*P*<0.001) but in group 4, GSPE administration significantly (*P*<0.001) reduced urine volume and urinary protein excretion as compared to Group 3. There was no significant difference between the two groups 1 and 2 *(P>0.05).*

Assessment of biochemical factors at the end of the 4-week period showed that the GSPE in group 4, significantly (*P*<0.001) decreased the elevated levels of FBS, BUN, Cr, TG, LDL, VLDL, Chol and increased the reduced levels TP, Alb and HDL levels in the group 3(*P*<0.005), However, no significant difference was observed between 1 and 2 *(P*>0.05*)* (Table 1).

**Table 1:** Different urine volume, urinary protein 24 hour and bichemical parameters**.**

**Groups**				**Parameters **
4	3	2	1	
24 ± 5.29 b 22.82 ± 4.10 b 153.33 ± 30.51^b^	65.83 ± 7.35 a 37.63 ± 1.88 a 282.16 ± 30.61^a^	9.66 ± 2.16 9.16 ± 2.34 93.16 ±10.43	9 ± 2.36 9.48 ± 2.51 96.16 ± 8.42	UV (mL) UP24h (mg/24h) FBS (mg.dl)
6.18 ± .83^b^	4.55 ± .51^a^	7.18 ± 1.04	7.44 ± .52	TP (g/dl)
4.42 ± .64^b^	2.30 ± .55^a^	4.96 ± 1.17	4.83 ± 1.31	Alb (g/dl)
29.46 ± 6.72^b^	46.94 ± 3.97^a^	15.79 ± 1.83	16.10 ± 3.73	BUN (mg/dl)
.50 ± .02^b^	.75 ± .09^a^	.43 ± .04	.42 ± .05	Cr (mg/dl)
93.38 ± 7.64^b^	135.78 ± 9.15^a^	67.57 ± 9.51	72.48 ± 7.53	TG (mg/dl)
122.06 ± 13.99^b^	188.76 ± 5.97^a^	82.47 ± 7.12	84.91 ± 4.48	Chol (mg/dl)
31.70 ± 3.08^b^	15.86 ± 2.43^a^	43.69 ± 6.18	41.23±3.56	HDL (mg/dl)
43.83 ± 4.38^b^	66.20 ± 5.94 a	26.79 ± 5.66	28.04 ± 3.83	LDL (mg/dl)
49.19 ± 9.96 b	106.69 ± 5.87 a	11.98 ± 4.9	13.33 ± 4.88	VLDL (mg/dl)

Each value is expressed as mean ± standard deviation (*n *= 6), differences at *p *< 0.05 were considered significant.

a – different than the healthy control group;

b – different than the diabetic control group.

*Changes in plasma MDA and erythrocyte antioxidant enzymes activities*

Table 2, demonstrates the differences in plasma MDA and erythrocyte SOD, GPx and CAT activities. A significant decrease *(p*<0.001) of plasma MDA and significant increase in erythrocyte GPx, CAT and SOD were observed in group 4 after administration GSPE as compared with group 3. Also group 2 showed no significant difference with group 1 *(P*>0.05).

**Table 2:** Effects of GSPE on oxidative stress and activities of antioxidant enzymes in plasma of experimental groups.

Groups					Parameters
4	3	2	1		
8.01 ± 0.55 b 2059.23 ± 90.34^b^ 157.07 ± 18.39 b	14.41 ± 0.6 a 1612.49 ± 68.28^a^ 93.24 ± 17.15^a^	6.85 ± 0.58 2107.8 ± 109.87 162.68 ± 27.38	7.22 ± 0.57 2149.03 ± 177.6 160.42 ± 23.61	MDA (nmol/mL) SOD (U/gHb) GPx (U/gHb)	
487.16 ± 48.16^b^	308.83 ± 45.51^a^	510.9 ± 41.42	496.59 ± 57.69		CAT (U/gHb)

Each value is expressed as mean ± standard deviation (*n *= 6), differences at *p *< 0.05 were considered significant.

a – different than the healthy control group;

b – different than the diabetic control group

## Discussion

STZ by increasing of free radicals formation and/or impaired antioxidant defense leads to generation of oxidative which can cause intensive injury in various tissues and may lead to various diseases such as diabetes (18). Antioxidants are substances which postpone or arrest the oxidation of cellular substrates that can be oxidized. Function of the different antioxidants is to eliminate superoxide, and/ or activating of detoxifying/defensive proteins (3). This study was designed to investigate the effect of GSPE on biochemical factors and antioxidant changes in STZ-induced diabetic rats. We observed that diabetic control rats developed severe polyuria as a result of osmotic diuresis. However, the diabetic rats that were treated with GSPE in group 4 showed significant decrease in urinary volume, probably as a result of the normalization of plasma glucose level or synergistic effect with insulin as shown in previous study (19, 20). Our study also showed, an increase UP24h in diabetic control rat. Proteinuria, a hallmark feature of early glomerular damage in patients with diabetes, is associated with renal hemodynamic and histologic changes (21) also increased synthesis of reactive oxygen species, or loss of nephrin in podocytes as shown by different investigators (22). GSPE-treated diabetic rats showed an impressive decrease in the amount of proteinuria like the previous study (23). Our findings indicate that FBS had a meaningful decrease in diabetic rats treated with GSPE as compared with diabetic control rats. It is likely that GSPE decreased glucose levels in diabetic rats by increasing circulating insulin levels (20). This findings supported by previous study (24). The results in Table 1 showed, reduction in plasma total protein and albumin level in diabetic control rats. The decrease in protein and albumin may be due to microproteinuria and albuminuria, and/or may be due to increased protein catabolism (25). Also we observed significant increase in the level of plasma BUN and Cr in the diabetic control rats when compared respective with healthy control group rats. Increased blood urea nitrogen and serum creatinine in diabetic rats indicates progressive renal damage (26) also increased urea nitrogen production in diabetes may be accounted for by enhanced catabolism of both liver and plasma proteins (27). In current study, serum levels of TG, LDL, VLDL and TC were significantly elevated in diabetic control rats when compared with healthy control group rats. Alterations in plasma lipoprotein metabolism are common in diabetes, which tend to exaggerate any pre-existing tendencies towards elevated lipid levels (28). In addition, diabetes is associated with increased dyslipidemia (29). Elevation of serum lipids indicate either the defective removal or overproduction (or both) of one or more lipoproteins (30). Administration of GSPE significantly improved all of these changes of biochemical factors caused by diabetes. Moreover, the serum level of HDL-C, which was significantly decreased in diabetic rats, was also improved by GSPE. These effects of GSPE are in agreement with the previous studies (20, 31, 32). Our results showed an increased MDA level in the plasma of diabetic control rats and a significantly decreased erythrocyte enzymatic antioxidant activities such as GPx, SOD and CAT. Hyperglycemia, reduces production and activities of some of antioxidant enzymes such as: SOD and GPx, likely by glycation (33). It is well known that people with diabetes have a lower antioxidant defense, enzymatic (SOD, catalase and GPx) and non-enzymatic (vitamin C, E or A, free radical scavengers). Also, increase of MDA information is probably due to the additional production of divers radical species. These radicals have been suggested to stimulate destruction of lipids and carbohydrates leading to hyperglycemia and related glucose auto-oxidation (34). All of these found changes in the antioxidant system and MDA were restored by the administration of GSPE. 

## Conclusion

We suggest that oral administration of GSPE provides a new and effective approach to attenuating metabolic disorders and defect of antioxidant system defense that induced by STZ. These effects are probably due to powerful antioxidant property of GSPE. Therefore, in view of these properties GSPE may be a good candidate to prevent the progression of diabetes complications.
